# Genetic characteristics of blastic plasmacytoid dendritic cell neoplasm: A single institution experience

**DOI:** 10.18632/oncotarget.28742

**Published:** 2025-06-17

**Authors:** Fei Fei, Milhan Telatar, Vanina Tomasian, Lisa Chang, Olga Danilova, Javier Arias-Stella, Raju Pillai, Lorinda Soma, Parastou Tizro, Pamela S. Becker, Anthony S. Stein, Guido Marcucci, Michelle Afkhami

**Affiliations:** ^1^Department of Pathology, City of Hope Comprehensive Cancer Center, Duarte, CA 91010, USA; ^2^Department of Hematology and Hematopoietic Cell Transplantation, City of Hope Comprehensive Cancer Center, Duarte, CA 91010, USA

**Keywords:** Blastic plasmacytoid dendritic cell neoplasm (BPDCN), Next-generation sequencing (NGS), *CCDC50*

## Abstract

Blastic plasmacytoid dendritic cell neoplasm (BPDCN) is a rare hematological malignancy with poorly characterized molecular features. To identify disease-specific mutational profiles, we performed targeted next-generation sequencing (NGS) on a cohort of 21 BPDCN patients. Our study revealed that *TET2* (57%) and *ASXL1* (33%) were the most frequently mutated genes, followed by *NRAS* (29%), *SRSF2* (14%), *ZRSR2* (14%), and *KMT2D* (14%). Further analysis demonstrated that poor prognosis was associated with older age (≥65 years), the presence of three or more mutations, *TET2* mutations, *TET2* truncating mutations, and mutations involving DNA methylation pathways. In contrast, patients who underwent hematopoietic stem cell transplantation (HSCT) exhibited more favorable clinical outcomes. Moreover, our study indicated that *CCDC50* expression was significantly elevated in BPDCN cases compared to those with acute myeloid leukemia (AML) or chronic monomyelocytic leukemia (CMML), suggesting that *CCDC50* may serve as a reliable diagnostic marker for distinguishing BPDCN from AML, as well as a potential biomarker for disease monitoring. Finally, our investigation of mutational profiles in sequentially paired specimens revealed a high prevalence of bone marrow clonal hematopoiesis in patients with BPDCN. In conclusion, the genetic landscape of BPDCN identified in this study provides valuable insights that may improve diagnostic accuracy and guide prognostic evaluation and therapeutic strategies. However, validation in larger, independent cohorts are warranted.

## INTRODUCTION

Blastic plasmacytoid dendritic cell neoplasm (BPDCN) is a rare hematological malignancy characterized by the proliferation of immature cells with plasmacytoid dendritic cell (pDC) differentiation [1]. Most patients are diagnosed after the age of 60 with a male predominance (male-to-female ratio: 4–6:1) [2–8]. Clinically, BPDCN typically presents with widespread involvement of multiple sites, including skin, bone marrow, peripheral blood, lymph nodes, spleen, and other organs [9]. Although the introduction of the CD123-targeted agent tagraxofusp and the use of allogeneic hematopoietic stem cell transplantation (HSCT) have led to improved clinical outcomes, the overall prognosis remains poor, with median overall survival (OS) ranging from approximately 8.7 to 24 months [4, 5, 10–13].

Currently, the diagnosis of BPDCN relies on a comprehensive evaluation of both morphological features and immunophenotypic characteristics. BPDCN cells typically express CD123, CD4, CD56, HLA-DR, TCL1, and TCF4, while lacking strong expression of lineage-specific markers for either lymphoid or myeloid cells [1]. Differentiating BPDCN from acute leukemia, particularly certain subsets of acute myeloid leukemia (AML), can be challenging due to overlapping clinical, morphological, and immunophenotypic features. In addition, BPDCN must be distinguished from mature pDC proliferations in patients with myeloid neoplasms [14–18].

Previous studies have demonstrated that recurrent mutations in genes involved in epigenetic regulation, such as *TET2* and *ASXL1*, are present in more than half of BPDCN cases [2, 19–23]. Additionally, mutations affecting the RNA splicing pathways, including *ZRSR2*, *SRSF2*, *U2AF1*, and *SF3B1*, are frequently observed in patients with BPDCN [22]. However, these genetic features, which substantially overlap with those observed in other myeloid neoplasms, do not provide diagnostic specificity for BPDCN. Moreover, the prognostic implications of these mutations in BPDCN remain poorly understood and warrant further investigation.

This study aims to characterize the genetic landscape of BPDCN using comprehensive next-generation sequencing (NGS) panels, with the goal of identifying molecular signatures that may have diagnostic and prognostic significance in clinical practice.

## RESULTS

### Case cohort characteristics

A total of 21 patients with a confirmed diagnosis of BPDCN who underwent NGS assays on bone marrow (*n* = 19), peripheral blood (*n* = 1), and skin (*n* = 1) specimens were included in our cohort. As shown in Table 1, our cohort consisted of 19 males (90%) and 2 females (10%), with a median age of 70 years (range: 12–88 years). The most prevalent ethnicity was White (16/21; 76%), followed by Asian (3/21; 14%) and Black or African American (1/21; 5%). All patients had bone marrow involvement by BPDCN, with tumor content ranging from less than 5% to 91% (mean: 44%). Aside from the bone marrow, the most frequently affected sites were skin (14/21; 67%) and lymph nodes (11/21; 52%), followed by central nervous system (5/21; 24%). Three patients had a history of prior or concurrent hematological malignancies, including myelodysplastic syndrome (MDS), myeloproliferative neoplasm (MPN), and classic Hodgkin lymphoma. The clinical and pathological features of these patients are summarized in Table 1 and Supplementary Table 1.

**Table 1:** Clinical and laboratory data of patients with BPDCN (*n* = 21)

Parameter	Values
**Age (years); (Median, range)**	70 (12–88)
**Sex (*n*, %)**	
**Male**	19 (90%)
**Female**	2 (10%)
**Ethnicity (*n*, %)**	
**White**	16 (76%)
**Asian**	3 (14%)
**Black or African American**	1 (5%)
**Unknown**	1 (5%)
**Involvement of Hematological Sites (*n*, %)**	
**Bone Marrow**	21 (100%)
**%Neoplastic cells in BM (Mean, Range)**	44 (<5–91%)
**Lymph node**	11 (52%)
**Spleen**	3 (14%)
**Liver**	3 (14%)
**Involvement of Extrahematological Sites (*n*, %)**	
**Skin**	14 (67%)
**CNS**	5 (24%)
**Other**	4 (19%)
**Prior or Concurrent Hematological Malignancy (*n*, %)**	3 (14%)
**Myelodysplastic Syndrome**	1 (5%)
**Myeloid Proliferative Neoplasm**	1 (5%)
**Classic Hodgkin Lymphoma**	1 (5%)
**Hematologic parameters at the time of initial BM biopsy**	
**WBC (K/uL), Average (Range)**	8.8 (0.4–55.3)
**RBC (M/uL), Average (Range)**	3.3 (2.2–5.0)
**Hb (g/dL), Average (Range)**	10.2 (7.6–15.9)
**Platelet (k/uL), Average (Range)**	97.1 (16–270)
**Karyotype Analysis (n, %)**	
**Normal**	9 (43%)
**Abnormal**	11 (52)
**Complex Karyotype (≥3 abnormalities)**	10 (48%)
**Unknown**	1 (5%)
**Hematopoietic Stem Cell Transplantation (*n*, %)**	
**Yes**	9 (43%)
**No**	12 (57%)
**Clinical Outcome (*n*, %)**	
**Alive**	7 (33%)
**Deceased**	14 (67%)
**Overall Survival (days); (Median, Range)**	415 (29–2920)

Abbreviations: BPDCN: Blastic plasmacytoid dendritic cell neoplasm; BM: Bone marrow; CNS: Central nervous system; Hb: Hemoglobin; RBC: Red blood cell; WBC: White blood cell.

### Cytogenetic findings

Previous studies have indicated that cytogenetic abnormalities are common in patients with BPDCN, with approximately 60% exhibiting complex karyotypes [2, 24]. Consistent with these findings, our study identified abnormal karyotypes in 11 of 20 patients with available cytogenetic studies (55%), including 10 patients (50%) with complex karyotypes, defined as ≥3 abnormalities (Supplementary Table 1). Further analysis revealed that chromosomal losses were the most common abnormalities, including −9 (10/20; 50%), −13 (5/20; 25%), −15 (5/20; 25%), and −1q (3/20; 15%). Chromosomal gains were less common, including +9p (2/20; 10%), +10p (2/20; 10%), and +19 (2/20; 10%). The most frequently observed structural rearrangements were translocations t(1;6)(q21;q23) and t(1;6)(q21;q25), each identified in three patients (15%). Reflex fluorescence *in situ* hybridization (FISH) was performed on a subset of cases, revealing *ETV6* deletions in five of nine cases and *TP53* deletions in three of nine cases. Prior studies have identified deletions involving 12p12/*ETV6* as among the most common cytogenetic findings in BPDCN, potentially representing early clonal events in disease evolution [2, 25, 26]. Additionally, *CDKN2A* deletions*, ERG1* deletions, monosomy *9,* and monosomy 13 were each observed in two cases. A *MYC* rearrangement was identified in one case.

### Mutation profiles in BPDCN patients

**Figure 1:**
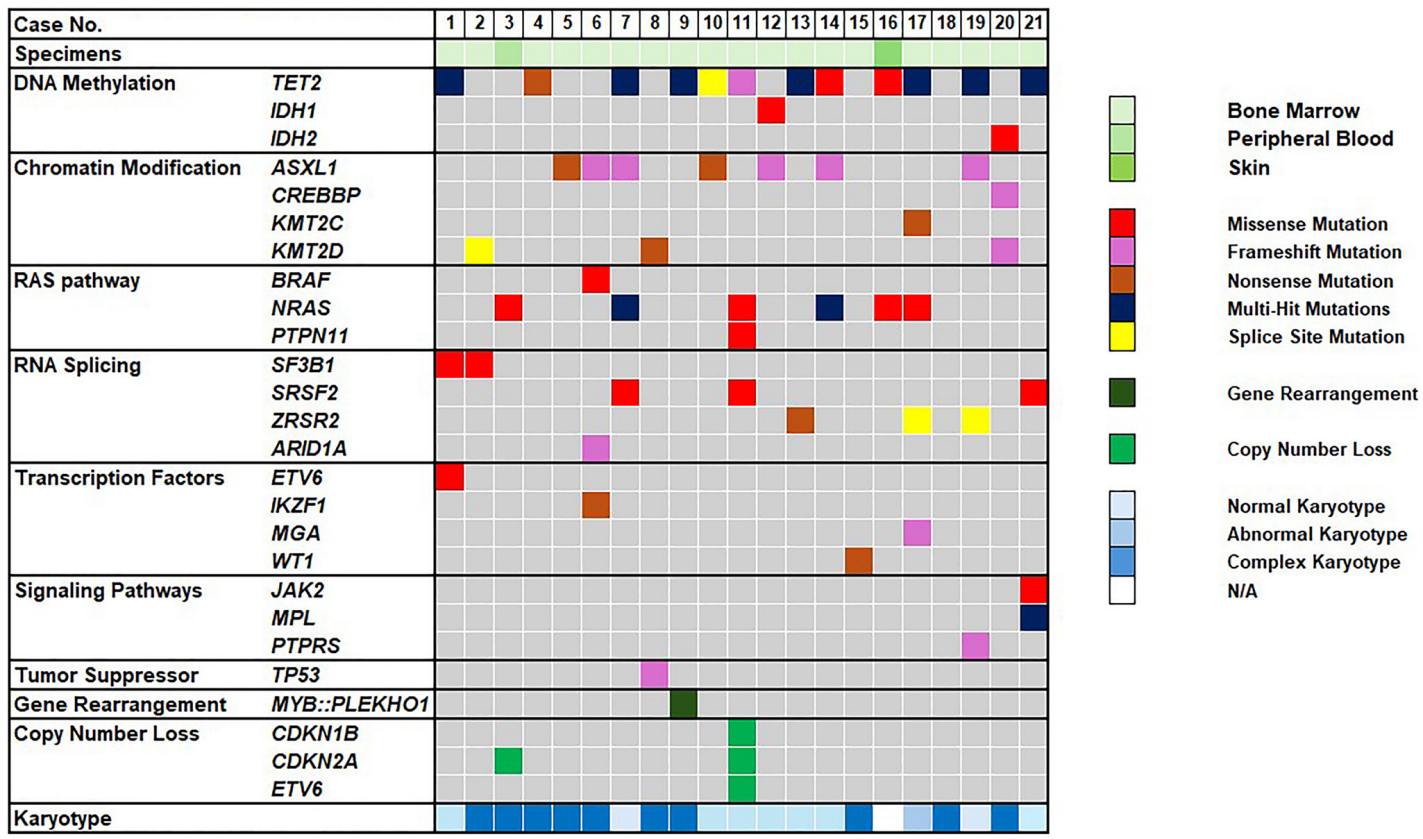
Genetic and cytogenetic features of 21 BPDNC patients. The Oncoplot illustrates the distribution of pathogenic/likely pathogenic mutations, gene rearrangements, copy number variations, and cytogenetic features in patients with BPDCN. Each column represents an individual patient. A total of 22 genes are categorized into seven functional groups: DNA methylation pathway, chromatin modification, RAS pathway, RNA splicing pathway, transcription factors, signaling pathways and tumor suppressors. Green indicates different specimen types: bone marrow, peripheral blood and skin. Cytogenetic findings are classified into four groups: normal karyotype, abnormal karyotype, complex karyotype (≥3 abnormalities) and N/A (not applicable).

As illustrated in Figure 1, pathogenic or likely pathogenic mutations were identified in 20 of 21 patients (95%), with 10 patients (48%) harboring three or more mutations. The most frequently mutated genes were *TET2* (12/21; 57%) and *ASXL1* (7/21; 33%), followed by *NRAS* (6/21; 29%), *SRSF2* (3/21; 14%), *ZRSR2* (3/21; 14%) and *KMT2D* (3/21; 14%). *TET2* mutations included nine frameshift mutations (9/21; 43%), seven missense mutations (7/21; 33%), three splice-site mutations (3/21; 14%), and two nonsense mutations (2/21; 10%), with a median variant allele frequency (VAF) of 36% (range: 3–46%). Multi-hit *TET2* mutations were observed in 7 of 12 cases (58%). *ASXL1* mutations included five frameshift mutations (5/7; 71%) and two nonsense mutations (2/7; 29%), with a median VAF of 26% (range: 5–32%). Notably, the *ASXL1* p.G646Wfs*12 mutation was recurrently identified in four cases. These findings are consistent with previous studies. [2, 19, 27, 28]. Non-recurrent mutations, including *IDH1*, *IDH2*, *CREBBP*, *KMT2C*, *BRAF*, *PTPN11*, *ARID1A*, *ETV6*, *IKZF1*, *MGA*, *WT1*, *JAK2*, *MPL*, *PTPRS*, and *TP53* were each identified in a single patient. Interestingly, a *MYB*::*PLEKHO1* gene rearrangement was identified in one patient, a finding that has been frequently reported in BPDCN cases [27, 29]. Additionally, concurrent *JAK2* and *MPL* mutations were detected in one patient whose bone marrow studies exhibited features consistent with MPN. To better identify potential founder mutations, Table 2 summarizes the detected mutations along with their corresponding VAFs and tumor percentages for each case.

**Table 2:** Genetic characteristics in BPDCN patients (*n* = 21).

Case No.	Specimen	Tumor %^*^	Mutations		Gene rearrangement	Copy number loss
			Genomic alterations	Allele frequency		
1	BM	42%	TET2 (c.2305del; p.Q769Sfs^*^44) SF3B1 (c.1988C>T; p.T663I) ETV6 (c.1105C>T; p.R369W) TET2 (c.5776_5804delinsTAG; p.R1926^*^)	34% 26% 12% 7%	N/A	N/A
2	BM	84%	KMT2D (c.14251+1G>A) SF3B1 (c.2098A>G; p.K700E)	44% 44%	N/A	N/A
3	PB	44%	NRAS (c.35G>A; p.G12D)	18%	N/A	CDKN2A
4	BM	88%	TET2 (c.2911G>T; p.E971^*^)	40%	N/A	N/A
5	BM	16%	ASXL1 (c.1249C>T; p.R417^*^)	5%	N/A	N/A
6	BM	86%	ARID1A (c.4404dup; p.G1469Wfs^*^22) ASXL1 (c.1934dup; p.G646Wfs^*^12) BRAF (c.1396G>A; p.G466R) IKZF1 (c.678C>A; p.Y226^*^)	40% 28% 38% 43%	N/A	N/A
7	BM	16%	ASXL1 (c.1900_1922del; p.E635Rfs^*^15) NRAS (c.190T>G; p.Y64D) NRAS (c.38G>T; p.G13V) SRSF2 (c.284C>A; p.P95H) TET2 (c.5390del; p.L1797Yfs^*^23) TET2 (c.3866G>T; p.C1289F)	26% 14% 4% 35% 41% 23%	N/A	N/A
8	BM	89%	KMT2D (c.15061C>T; p.R5021^*^) TP53 (c.390_426del; p.N131Cfs^*^27)	43% 72%	N/A	N/A
9	BM	80%	TET2 (c.2478_2479dup; p.A827fs^*^15) TET2 (c.4044+1G>A)	45% 36%	MYB::PLEKHO1	N/A
10	BM	7.50%	ASXL1 (c.2338C>T; p.Q780^*^) TET2 (c.3803+2T>G)	20% 16%	N/A	N/A
11	BM	40%	NRAS (c.35G>C; p.G12A) PTPN11 (c.226G>A; p.E76K) SRSF2 (c.284C>T; p.P95L) TET2 (c.3088del; p.Q1030fs^*^3)	4% 3% 37% 41%	N/A	CDKN1B CDKN2A ETV6
12	BM	5%	ASXL1 (c.1934dup; p.G646Wfs^*^12) IDH1 (c.395G>A;p.R132H)	23% 14%	N/A	N/A
13	BM	6.50%	TET2 (c.4532T>A; p.L1511^*^) TET2 (c.3965T>A; p.L1322Q) ZRSR2 (c.883C>T; p.R295^*^)	37% 30% 28%	N/A	N/A
14	BM	85%	ASXL1(c.1934dup; p.G646Wfs^*^12) NRAS (c.35G>A; p.G12D) NRAS (c.187G>A; p.E63K) TET2 (c.5618T>C; p.I1873T)	27% 7% 35% 46%	N/A	N/A
15	BM	91%	WT1 (c.1372C>T; p.R458^*^)	47%	N/A	N/A
16	LN	90%	NRAS (c.35G>T; p.G12V) TET2 (c.4159A>C; p.N1387H)	43% 45%	N/A	N/A
17	BM	65%	KMT2C (c.997C>T; p.Q333^*^) MGA (c.7592dup; p.K2532Efs^*^6) NRAS (c.181C>A; p.Q61K) TET2 (c.822del; p.N275fs^*^18) TET2 (c.5711A>G; p.H1904R) TET2 (c.3443A>G; p.Y1148C) ZRSR2 (c.827+1G>A)	6% 7% 19% 3% 34% 37% 67%	N/A	N/A
18	BM	40%	N/A	N/A	N/A	N/A
19	BM	70%	ASXL1 (c.1934dup; p.G646Wfs^*^12) PTPRS (c.3820dup; p.Q1274Pfs^*^40) TET2 (c.3075dup; p.E1026^*^) TET2 (c.3646C>T; p.R1216^*^) TET2 (c.4045-1G>T) ZRSR2 (c.312+1G>A)	32% 37% 30% 12% 43% 13%	N/A	N/A
20	BM	32%	CREBBP (c.5830dup; p.A1944Gfs^*^22) IDH2 (c.418C>T; p.R140W) KMT2D (c.10369_10370del; p.L3457Afs^*^10)	2% 29% 17%	N/A	N/A
21	BM	5%	MPL (c.79+2T>A) SRSF2 (c.284C>A; p.P95H) TET2 (c.4433del; p.K1478Sfs^*^93) MPL (c.1543T>A; p.W515R) JAK2 (c.2047A>G; p.R683G) TET2 (c.3640C>T; p.R1214W)	46% 43% 40% 39% 14% 13%	N/A	N/A

^*^Tumor % was detected by the corresponding flow cytometry studies. Abbreviations: BM: Bone marrow; BPDCN: Blastic plasmacytoid dendritic cell neoplasm; LN: lymph node; N/A: Not applicable; PB: Peripheral blood.

### Prognostic analysis

**Figure 2:**
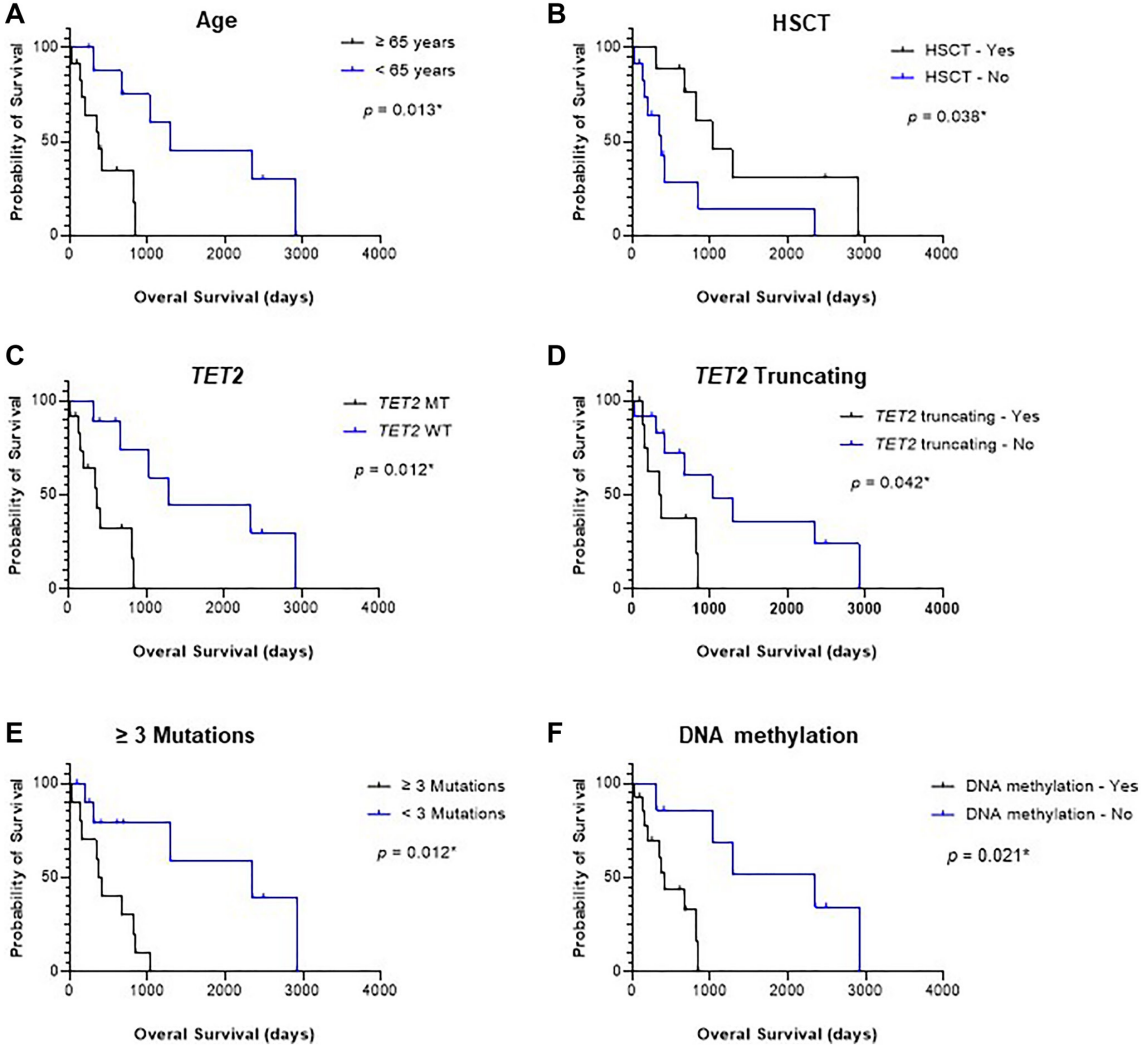
Overall survival (OS) analysis based on clinical and genetic factors (*n* = 21). (**A**) Patients aged ≥65 years demonstrated significantly shorter OS compared to those younger than 65 years (*p* = 0.013^*^). (**B**) Patients who underwent hematopoietic stem cell transplantation (HSCT) exhibited improved OS compared to those who did not receive HSCT (*p* = 0.038^*^). (**C**) Patients harboring *TET2* mutations had significantly worse OS than those without *TET2* mutations (*p* = 0.012^*^). (**D**) Patients with *TET2* truncating mutations showed inferior OS compared to those without such mutations (*p* = 0.042^*^). (**E**) Patients with three or more mutations experienced poorer clinical outcomes compared to those with fewer than three mutations (*p* = 0.012^*^). (**F**) Mutations affecting the DNA methylation pathway were associated with significantly reduced OS compared to patients without such mutations (*p* = 0.021^*^). A *p*-value of < 0.05 was considered statistically significant (^*^).

The overall prognosis for patients with BPDCN remains remarkably poor. In our cohort, the median OS was 415 days (range: 29–2920 days). To identify significant prognostic factors associated with OS, both univariate and multivariate analyses were performed (Table 3). Univariate analysis revealed that age over 65 years (*p* = 0.013) was associated with worse prognosis, while patients who underwent HSCT demonstrated prolonged OS (p = 0.038) (Figure 2A, 2B). Additionally, we found that patients harboring *TET2* mutations (*p* = 0.012), *TET2* truncating mutations (*p* = 0.042), or the presence three or more mutations (*p* = 0.012) exhibited significantly reduced OS (Figure 2C–E). Next, we categorized genes according to their functional pathways and analyzed their correlation with clinical outcomes. Interestingly, mutations affecting the DNA methylation pathway (*p* = 0.021) were associated with poorer clinical outcomes (Figure 2F). In contrast, no significant association was observed between OS and other factors, including gender, karyotype abnormalities, *ASXL1* or *NRAS* mutations, or mutations involving histone modification or RNA splicing pathways. Notably, only potential founder mutations were included in the univariate and multivariate analyses. However, multivariate analysis did not identify any independent prognostic factors significantly correlated with OS, likely due to the limited sample size in our study.

**Table 3:** Univariate and multivariate analyses of clinical and genetic characteristics among patients with BPDCN

Variable	Univariate (OS)		Multivariate (OS)	
	HR (95% CI)	*p*-value	HR (95% CI)	*p*-value
**Age (≥ 65 vs. < 65)**	7.441 (1.530–36.194)	0.013^*^	3726460.675 (0.000–4.852E+79)	0.86
**Gender (Female vs. Male)**	0.465 (0.059–3.661)	0.467	0.027 (0.000–3.318)	0.141
**Transplantation (No vs. Yes)**	3.283 (1.067–10.104)	0.038^*^	1.098 (0.117–10.266)	0.935
**Cytogenetics (Abnormal vs. Normal)**	0.334 (0.095–1.173)	0.087	2.615 (0.035–197.711)	0.663
**Mutations (≥ 3 vs. <3)**	7.198 (1.544–33.566)	0.012^*^	3873527198.8 (0.000–1.740E+95)	0.826
***TET2* (MT vs. WT) **	7.449 (1.552–35.759)	0.012^*^	3138378297.5 (0.000–6.342E+115)	0.861
***TET2* Truncation Mutation (Yes vs. No) **	3.654 (1.050–12.715)	0.042^*^	0.000 (0.000–7.973E+92)	0.944
***ASXL1* (MT vs. WT) **	0.730 (0.227–2.347)	0.597	1.246 (0.020–79.427)	0.917
***NRAS* (MT vs. WT) **	1.519 (0.466–4.950)	0.488	0.044 (0.001–2.291)	0.121
**DNA methylation pathway (Yes vs. No)**	12.157 (1.454–101.641)	0.021^*^	0.000 (0.000–9.194E+75)	0.824
**Histone modification pathway (Yes vs. No)**	1.061 (0.362–3.113)	0.914	15.084 (0.423–538.232)	0.137
**RNA splicing pathway (Yes vs. No)**	3.056 (0.902–10.353)	0.073	0.000 (0.000–6.200E+74)	0.802

^*^Indicates statistical significance (*p* < 0.05) in predicting overall survival. Abbreviations: BPDCN: Blastic plasmacytoid dendritic cell neoplasm; CI: Confidence interval; HR: Hazard ratio; MT: Mutation; OS: Overall survival; WT: Wild type.

### *CCDC50* is highly expressed in BPDCN than AML and CMML

**Figure 3:**
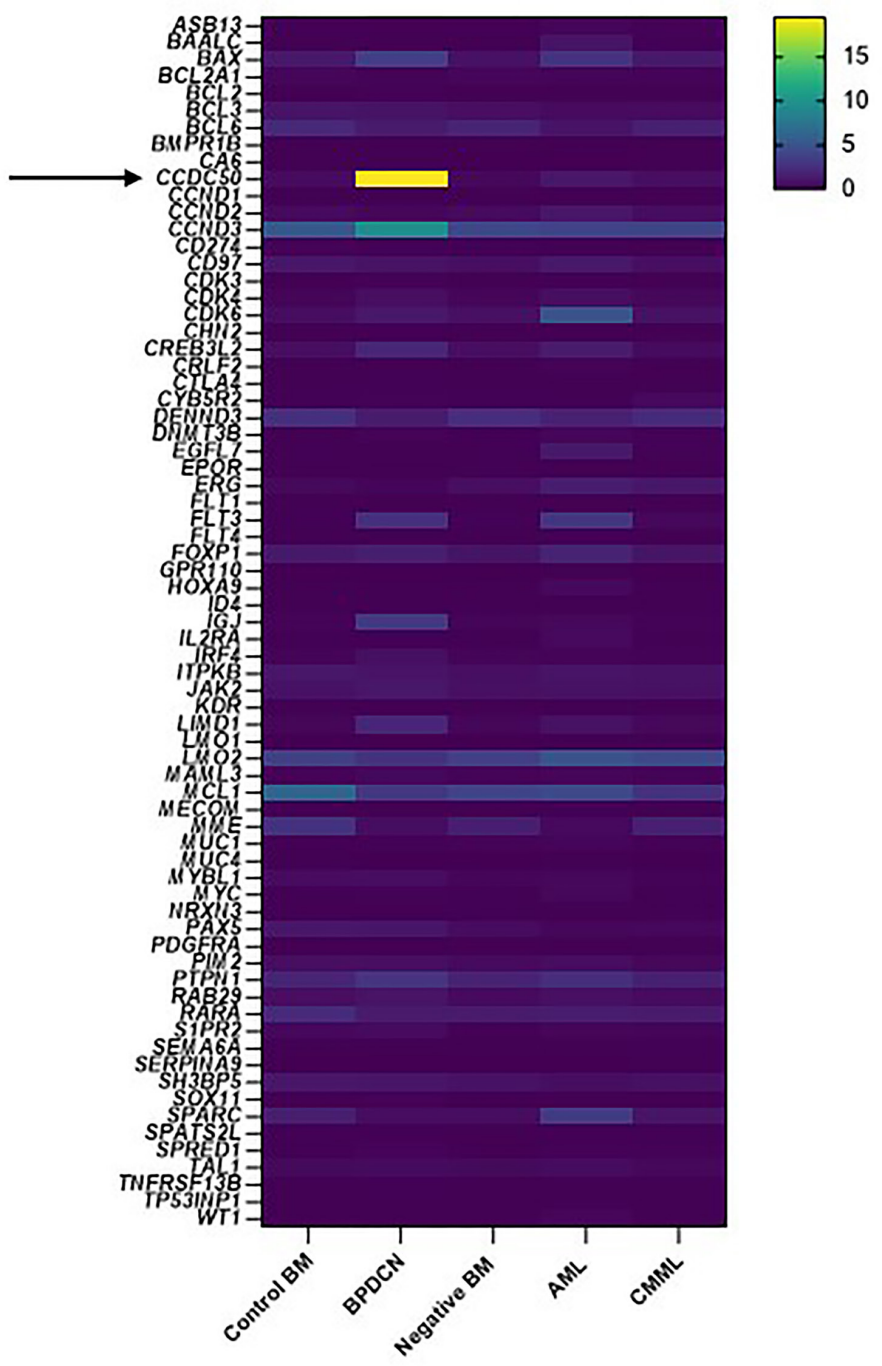
RNA expression profiling of 71 genes associated with hematological malignancies in patients with BPDCN, AML and CMML. The heatmap demonstrates a significant upregulation of *CCDC50* expression in BPDCN compared to AML and CMML. Control BM (*n* = 10): bone marrow aspirates without evidence of hematological malignancy; BPDCN (*n* = 6): bone marrow aspirates with confirmed BPDCN involvement; Negative BM (*n* = 10): bone marrow aspirates from patients with a history of BPDCN but without current involvement; AML (*n* = 10): bone marrow aspirates with AML involvement; CMML (*n* = 10): bone marrow aspirates with CMML involvement. Upregulated genes are shown in shades ranging from light green to yellow.

**Figure 4:**
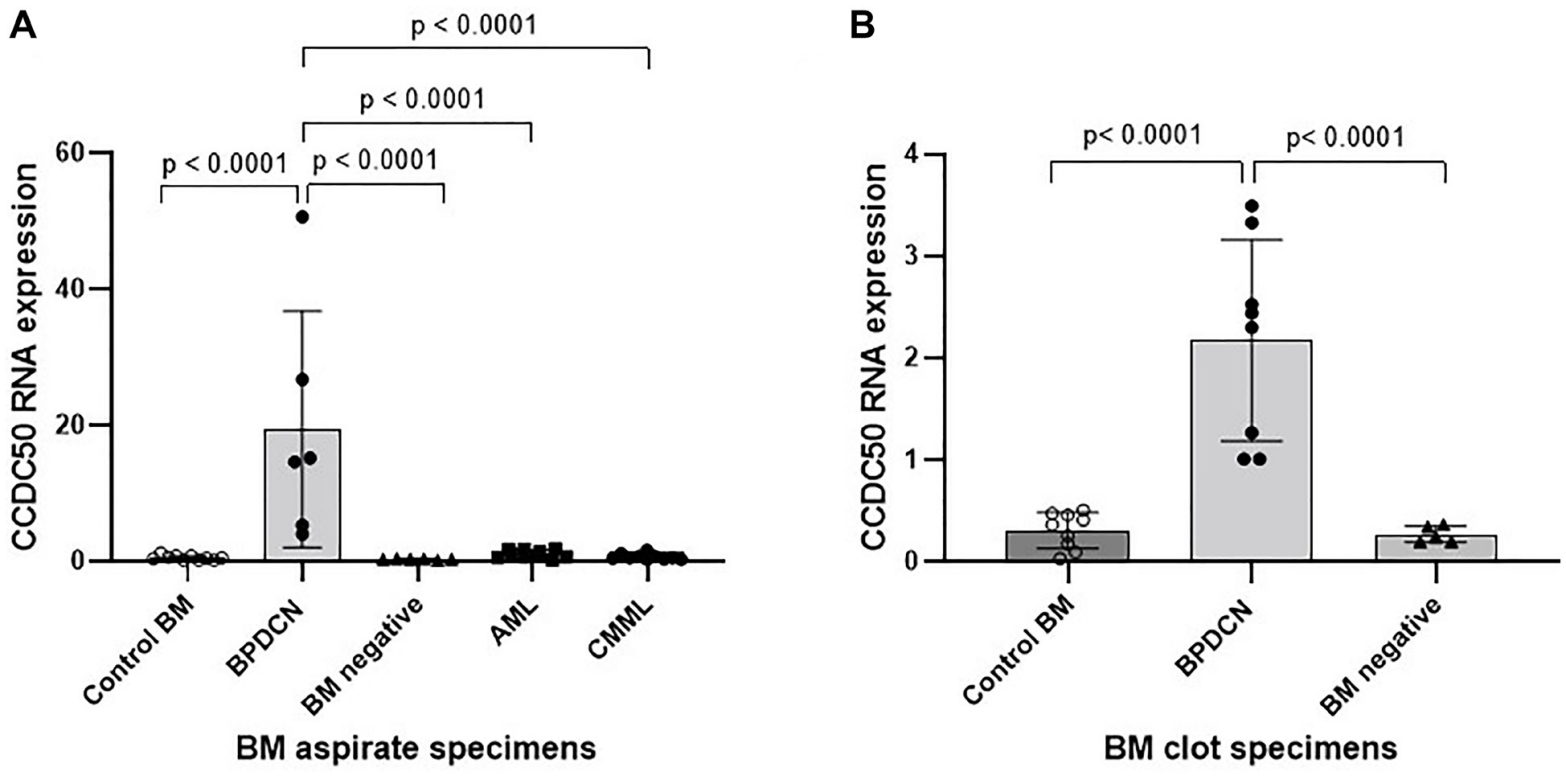
CCDC50 expression is significantly upregulated in BPDCN patients. (**A**) *CCDC50* expression is significantly higher in bone marrow aspirates from BPDCN patients compared to those with AML or CMML (*p* < 0.0001). Control BM (*n* = 10): bone marrow aspirates without evidence of hematological malignancy; BPDCN (*n* = 6): bone marrow aspirates with confirmed BPDCN involvement; Negative BM (*n* = 10): bone marrow aspirates from patients with a history of BPDCN but without current involvement; AML (*n* = 10): bone marrow aspirates with AML involvement; CMML (*n* = 10): bone marrow aspirates with CMML involvement. (**B**) *CCDC50* expression is also significantly elevated in bone marrow clot sections from BPDCN patients (*p* < 0.0001). Control BM (*n* = 9): bone marrow clot sections without evidence of hematological malignancy; BPDCN (*n* = 8): bone marrow clot sections with confirmed BPDCN involvement; Negative BM (*n* = 5): bone marrow clot sections from patients with a history of BPDCN but without current involvement.

To identify potential biomarkers specific to BPDCN, we analyzed the RNA expression profiles of 71 genes associated with hematological malignancies in patients with BPDCN, AML and chronic monomyelocytic leukemia (CMML). Among these genes, *CCDC50* was significantly upregulated in BPDCN, as illustrated in Figure 3. Subsequent comparative analysis revealed that *CCDC50* expression was markedly elevated in BPDCN compared to both AML (*p* < 0.0001) and CMML (*p* < 0.0001) (Figure 4A). However, *CCDC50* expression levels were relatively lower in clot sections compared to bone marrow aspirates (Figure 4B). These findings are consistent with those reported by Beird et al., who also demonstrated higher *CCDC50* expression in BPDCN compared to AML [30].

### Genetic features in BPDCN patients with sequential specimens

In the next step, we compared the genetic features of five patients with sequential specimens (Supplementary Table 2). Khanlari et al. previously reported that certain mutations identified in BPDCN patients may originate from bone marrow hematopoietic cells [31]. In Cases 2, 4, and 5, although bone marrow studies showed no evidence of BPDCN involvement, specific mutations persisted with VAFs similar to those observed in prior specimens with confirmed BPDCN involvement. In case 4, the presence of *ASXL1* p.G646fs*12 and *IDH1* p.R132H mutations suggests clonal hematopoiesis, as these mutations were eradiated following HSCT. In contrast, the mutational profile in Case 5 indicated that *MGA* p.K2532Efs*6, *NRAS* p.Q61K, and *TET2* p.Y1148C mutations were specific to BPDCN, as they were no longer detectable when bone marrow studies showed no evidence of disease. These findings suggest that although the bone marrow findings did not meet the WHO criteria for MDS or MPN, the underlying biology may resemble clonal cytopenia of undetermined significance (CCUS). Interestingly, we observed that *CCDC50* expression could serve as a valuable biomarker for monitoring BPDCN, as its levels were markedly decreased in Cases 3, 4, and 5 when there was no evidence of BPDCN involvement in the bone marrow.

## DISCUSSION

BPDCN is a rare hematological malignancy, and its genetic characteristics remain poorly understood. In this study, we investigated the mutational profiles and its clinical implications in 21 patients with BPDCN using comprehensive NGS assays. Our findings demonstrate that the mutational landscape of BPDCN is similar to that observed in other myeloid neoplasms, and we identified *CCDC50* as a potential biomarker for this disease. Furthermore, our study highlights prognostic factors associated with clinical outcomes and reinforces the critical role of clonal hematopoiesis in the pathogenesis of BPDCN.

In this study, we characterized the mutational landscape and associated clinical features of 21 patients with BPDCN using comprehensive NGS assays. Consistent with prior studies, *TET2* (57%) and *ASXL1* (33%) were the most frequently mutated genes in our cohort, with the majority of these being truncating mutations [2, 25, 28]. Similar findings were reported by Yin et al., who identified *TET2* and *ASXL1* mutations in 56% and 46% of BPDCN cases, respectively, in a cohort of 50 patients [2]. Their findings support the hypothesis that *TET2* and *ASXL1* mutations may represent early events in BPDCN pathogenesis, while additional mutations are likely acquired secondarily through genomic instability and clonal evolution driven by epigenetic dysregulation. While copy number loss at the *TP53* locus was observed in several cases, a *TP53* mutation was identified in only one patient. Interestingly, genes commonly mutated in AML and other myeloid neoplasms, such as *FLT3*, *NPM1*, *KIT*, *RUNX1*, and *DNMT3A*, were absent in our cohort. However, mutations in the RAS signaling pathway, including *NRAS*, *PTPN11*, and *BRAF*, were detected in 33% of cases (7/21), highlighting the potential for therapeutic strategies targeting the RAS signaling pathway in BPDCN. Furthermore, we identified mutations in genes involved in RNA splicing pathways, with a cumulative frequency of 38% (8/21). These findings are in concordance with those reported by Yin et al., who observed a collective mutation frequency of 23% in RNA splicing factor genes [2]. Similarly, Summerer et al. reported recurrent mutations in *SRSF2* (7/21; 33%), *SF3B1* (2/21; 10%), *U2AF1* (2/21; 10%), and *ZRSR2* (2/21; 10%) through the analysis of 1367 mutations across 1210 genes in 21 BPDCN cases, [32]. In a separate study, Renosi et al. using a 68-gene NGS panel in 13 BPDCN cases, identified mutations in *ZRSR2* and *SRSF2* in 31% and 15% of cases, respectively [20]. These findings support prior evidence suggesting that mutations in splicing factor genes may contribute to BPDCN pathogenesis by disrupting pathways critical for dendritic cell maturation and activation, thereby promoting tumorigenesis [25, 32, 33].

Prognostic factors in BPDCN remain controversial, largely due to the rarity of the disease. Our findings revealed that older age (≥65 years), *TET2* mutations, *TET2* truncating mutations, the presence of more than three mutations, and mutations affecting DNA methylation pathways were associated with a poorer prognosis. In contrast, patients who underwent HSCT demonstrated improved clinical outcomes. Consistent with our findings, Yin et al. reported that older age, mutations in genes associated with DNA methylation, and the presence of more than three mutations were associated with a poorer prognosis in a cohort of 50 BPDCN patients. In contrast, patients who underwent HSCT showed improved clinical outcomes [2]. However, Summerer et al., in their analysis of 21 BPDCN cases, did not identify significant differences in OS based on mutational patterns, copy number variations, or gene expression profiles [32]. Conversely, Beird and colleagues, in a cohort of 57 BPDCN patients, found that OS was significantly worse among individuals harboring at least one truncating *TET2* mutation [34]. Similarly, Khanlari et al., in a study of 51 BPDCN patients, demonstrated that the presence of bone marrow clonal hematopoiesis, including cases with *TET2* mutations, was associated with significantly inferior survival compared to those without such findings [31]. Additionally, a systematic literature review of 74 BPDCN cases by Gong et al. indicated that younger age and HSCT were significant factors associated with better clinical outcomes in BPDCN patients [35]. Similarly, Lin et al., analyzing data from the Surveillance, Epidemiology, and End Results (SEER) database, which included 697 BPDCN patients, identified older age and male gender as independent risk factors for OS based on multivariate Cox regression [36]. We believe that discrepancies among these studies may, in part, be attributable to the limited sample sizes in each cohort.

Furthermore, our RNA sequencing assay identified *CCDC50* as being highly expressed in BPDCN compared to AML and CMML. *CCDC50* is ubiquitously expressed across various tissues, and previous studies have demonstrated its role in promoting cell survival and NF-κB inducibility in mantle cell lymphoma and chronic lymphocytic leukemia [37]. However, the biological significance of *CCDC50* expression in BPDCN remains largely unexplored. Consistent with our findings, Beird et al. demonstrated that the plasmacytoid dendritic cell markers *CCDC50* and *LAMP5* were significantly more highly expressed in BPDCN than in AML, as revealed by transcriptome microarray analysis [30]. This finding indicates the dendritic cell origin of BPDCN. Additionally, prior studies have shown that *CCDC50* can suppress the ligand-mediated downregulation of the epidermal growth factor receptor (EGFR) and act as a multifunctional regulator in NF-κB and Fas signaling pathways [38]. By comparing the genetic features of BPDCN patients with sequential paired specimens, we observed that *CCDC50* expression serves not only as a reliable marker for distinguishing BPDCN from AML in diagnostically challenging cases but also as an effective indicator for disease monitoring. For instance, in Case 3 and 4, bone marrow studies showed no evidence of BPDCN, yet certain myeloid-related mutations, such as *ASXL1*, *IDH1* and *TET2*, persisted at relatively high VAFs. However, *CCDC50* expression levels were markedly decreased in these cases, supporting its potential as a disease-specific biomarker.

Approximately 10–20% of patients with BPDCN have been reported to have a history of, or develop post-treatment MDS, CMML, or AML, indicating a myeloid origin of the neoplastic clone [33, 39]. In our cohort, prior or concurrent hematological malignancies were identified in 14% (3/21) of patients, including MDS, MPN and classic Hodgkin lymphoma. Khanlari et al. through NGS analysis of multiple bone marrow specimens, skin lesions and sorted BPDCN cells, demonstrated that bone marrow clonal hematopoiesis is highly prevalent in elderly BPDCN patients [31]. Their findings suggest that some bone marrow specimens, while not meeting WHO criteria for MDS or CMML, biologically resemble clonal cytopenia of undetermined significance or low-grade MDS. Similarly, in our study, a comparative analysis of mutational profiles across paired specimens revealed that, even in cases without BPDCN bone marrow involvement or a concurrent diagnosis of myeloid neoplasms, mutations commonly associated with myeloid neoplasms persisted at relatively high VAFs. These mutations were only eliminated after HSCT.

Our study has several limitations. First, it is a retrospective study of only 21 BPDCN patients; therefore, the findings should be interpreted with caution and validated in larger, independent cohorts. Second, despite reviewing the VAFs for each gene, we were unable to definitively determine the founder mutations, given the high prevalence of bone marrow clonal hematopoiesis in elderly BPDCN patients. Third, pathological, molecular, and cytogenetic data were not available for all cases at the time of initial diagnosis.

In conclusion, our study investigated the mutational landscape of BPDCN and its association with clinical outcomes. Importantly, we demonstrate for the first time that *CCDC50* expression serves as a reliable diagnostic marker for distinguishing BPDCN from AML in diagnostically challenging cases, as well as an effective biomarker for disease monitoring. Furthermore, our findings confirm the high prevalence of bone marrow clonal hematopoiesis in patients with BPDCN, highlighting its potential role in disease pathogenesis. However, these findings should be interpreted with caution and validated in larger, independent cohorts.

## MATERIALS AND METHODS

### Patients and specimens

The study was approved by the Institutional Review Board of City of Hope Comprehensive Cancer Center. We performed a retrospective analysis of patients diagnosed with BPDCN who underwent NGS assays at the CLIA-approved Clinical Molecular Diagnostics Laboratory between April 2018 and October 2024. A total of 21 patients were identified, and their clinical, pathological, and molecular findings were collected through chart reviews.

### DNA-based NGS assay

The DNA-based NGS Heme panel encompasses 135 genes, as listed in Supplementary Table 3. These panels are designed to detect single-nucleotide variants (SNVs), insertions/deletions (indels), copy number variants (CNVs), and splice site variants. Peripheral blood, bone marrow aspirates, and bone marrow clot sections were used as input materials, with a minimum DNA requirement of 40 ng. The workflow includes acoustic shearing of isolated genomic DNA, followed by library preparation and targeted gene enrichment using a capture-based method. The normalized and enriched libraries are pooled, clustered onto flow cells, and sequenced using the Illumina NextSeq 550 platform. Sequencing data are subsequently analyzed using the Local Run Manager TruSight Oncology Comprehensive analysis module.

### RNA-based NGS assay

The RNA-based NGS assay utilizes the Archer platform to detect gene rearrangements across 165 genes relevant to hematological malignancies, solid tumors, and sarcomas. Additionally, it assesses RNA expression in 71 genes associated with hematological malignancies. The gene lists for the RNA-based NGS fusion and RNA expression panels are provided in Supplementary Table 4. Peripheral blood, bone marrow aspirates, and bone marrow clot sections were used as input materials, with a minimum RNA requirement of 200 ng. The workflow includes RNA extraction and library preparation using the Archer FusionPlex reagent kit for Illumina sequencing. The cDNA strands undergo end repair, adenylation, and ligation with half-functional universal adapters containing sample barcodes. Two rounds of multiplex polymerase chain reaction (PCR) are then performed using gene-specific primers (GSP1 and GSP2), along with a primer complementary to the universal adapter, to enrich target genes and enable the identification of both known and novel fusion partners. The target amplicon library is quantified and sequenced on the Illumina MiSeq system, with subsequent data analysis performed using Archer FusionPlex Software.

### Statistical analysis

Baseline characteristics are presented as medians or means and ranges for continuous variables and as frequencies for categorical variables. OS was defined as the time from diagnosis to the last follow-up or death from any cause. The Cox proportional hazards regression model was applied to identify significant factors influencing OS. A *p*-value of < 0.05 was considered statistically significant. Data analyses were performed using GraphPad Prism software (Version 9.3.1) or IBM SPSS Statistics (Version 29).

## SUPPLEMENTARY MATERIALS




