# Supplementary material for: Genetic characteristics of blastic plasmacytoid dendritic cell neoplasm: A single institution experience
Source: Oncotarget. 2025 Jun 17;16:495–507. doi: 10.18632/oncotarget.28742 (PMC12173198; doi:10.18632/oncotarget.28742)
Supplement: Supplementary file 1 [file oncotarget-16-28742-s001.pdf]

Genetic characteristics of blastic plasmacytoid dendritic cell neoplasm: A single institution experience

SUPPLEMENTARY MATERIALS

Supplementary Table 1: Clinical and pathological features of patients with BPDCN (*n* = 21). See Supplementary Table 1.

Supplementary Table 2: Genetic characteristics of BPDCN patients with sequential specimens (*n* = 5). See Supplementary Table 2.

Supplementary Table 3: List of genes in DNA-based next-generation sequencing heme panel

Entire coding region mutation analysis of 135 genes										
ABL1	*ARID1A*	*ASXL1*	*ATM*	*B2M*	*BCL2*	*BCL6*	*BCOR*	*BCORL1*	*BIRC3*	*BRAF*
BTK	*CALR*	*CARD11*	*CBL*	*CBLB*	*CCND1*	*CCND3*	*CD38*	*CD3E*	*CD3G*	*CD79A*
CD79B	*CDK4*	*CDK7*	*CDKN1B*	*CDKN2A*	*CDKN2B*	*CDKN2C*	*CEBPA*	*CHD2*	*CRBN*	*CREBBP*
CSF3R	*CUX1*	*CXCR4*	*DDx3x*	*DIS3*	*DNMT3A*	*E2F1*	*EGFR*	*EP300*	*ETV6*	*EZH2*
FBXW7	*FGFR3*	*FH*	*FLT3*	*FAM46C*	*FOXO1*	*GATA1*	*GATA2*	*GNA13*	*GNAS*	*HCK*
HRAS	*ID3*	*IDH1*	*IDH2*	*IGLL5*	*IKZF1*	*IL6*	*IL7R*	*IRF4*	*JAK1*	*JAK2*
JAK3	*KDM6A*	*KIT*	*KMT2A*	*KMT2C*	*KMT2D*	*KRAS*	*LCK*	*LMO2*	*MAPK1*	*MEF2B*
MGA	*MIR142*	*MPL*	*MYC*	*MYD88*	*NFKB2*	*NOTCH1*	*NOTCH2*	*NPM1*	*NRAS*	*NTRK1*
PAX5	*PDCD1*	*PDGFRA*	*PHF6*	*PIGA*	*PIK3CA*	*PIK3CD*	*PIK3CG*	*PIK3R5*	*PLCG2*	*POT1*
PRDM1	*PTEN*	*PTK2B*	*PTPN11*	*RAD21*	*RB1*	*RFC4*	*RPS15*	*RUNX1*	*SETBP1*	*SF3B1*
SMC1A	*SMC3*	*SOCS1*	*SOCS6*	*SPI1*	*SRSF2*	*STAG2*	*STAT3*	*STAT5A*	*STAT5B*	*STAT6*
SUZ12	*TCF3*	*TET2*	*TNFAIP3*	*TP53*	*TP63*	*U2AF1*	*UBR5*	*WHSC1*	*WHSC1L1*	*WT1*
XPO1	*ZEB1*	*ZRSR2*								

Supplementary Table 4A: List of genes in RNA-based fusion panel

RNA-based fusion panel: 165 genes														
ABL1	*ABL2*	*AKT3*	*ALK*	*ARHGAP26*	*AXL*	*BCL11B*	*BCL2*	*BCL6*	*BCOR*	*BCR*	*BIRC3*	*BRAF*	*BRD3*	*BRD4*
CAMTA1	*CBFB*	*CCNB3*	*CCND1*	*CCND3*	*CD151*	*CDK6*	*CHD1*	*CHIC2*	*CIC*	*CIITA*	*CREBBP*	*CRLF2*	*CSF1R*	*DEK*
DUSP22	*EBF1*	*EGFR*	*EIF4A1*	*EPC1*	*EPOR*	*ERG*	*ESR1*	*ESRRA*	*ETV1*	*ETV4*	*ETV5*	*ETV6*	*EWSR1*	*FGFR1*
FGFR2	*FGFR3*	*FGR*	*FOSB*	*FOXO1*	*FUS*	*GLI1*	*GLIS1*	*HMGA2*	*IKZF1*	*IKZF2*	*IKZF3*	*IL2RB*	*INSR*	*JAK2*
JAZF1	*KAT6A*	*KLF2*	*KMT2A*	*LMO2*	*LYN*	*MALT1*	*MAML2*	*MAN2B1*	*MAST1*	*MAST2*	*MBTD1*	*MEAF6*	*MECOM*	*MFE2D*
MET	*MGEA5*	*MKL1*	*MKL2*	*MLF1*	*MLLT10*	*MLTT4*	*MN1*	*MSMB*	*MUSK*	*MYB*	*MYC*	*MYH11*	*NCOA1*	*NCOA2*
NF1	*NFKB2*	*NOTCH1*	*NOTCH2*	*NPM1*	*NR4A3*	*NRG1*	*NTRK1*	*NTRK2*	*NTRK3*	*NUMBL*	*NUP214*	*NUP98*	*NUTM1*	*P2RY8*
PAG1	*PAX3*	*PAX5*	*PAX7*	*PBX1*	*PDCDILG2*	*PDGFB*	*PDGFRA*	*PDGFRB*	*PHF1*	*PICALM*	*PIK3CA*	*PKN1*	*PLAG1*	*PML*
PPARG	*PRDM16*	*PRKCA*	*PRKCB*	*PRKD1*	*PRKD2*	*PRKD3*	*PTK2B*	*RAF1*	*RANBP17*	*RARA*	*RBM15*	*RECK*	*RELA*	*RET*
ROS1	*RSPO2*	*RSPO3*	*RUNX1*	*RUNX1T1*	*SEMA6A*	*SETD2*	*SS18*	*STAG2*	*STAT6*	*STIL*	*TAF15*	*TAL1*	*TCF12*	*TCF3*
TERT	*TFE3*	*TFEB*	*TFG*	*THADA*	*TMPRSS2*	*TP63*	*TSLP*	*TYK2*	*USP6*	*YAP1*	*YWHAE*	*ZCCHC7*	*ZMYND11*	*ZNF384*

Supplementary Table 4B: List of genes in RNA expression panel

Expression analysis of 71 Genes						
ASB13	*BAALC*	*BAX*	*BCL2A1*	*BCL2*	*BCL3*	*BCL6*
BMPR1B	*CA6*	*CCDC50*	*CCND1*	*CCND2*	*CCND3*	*CD274*
CD97	*CDK3*	*CDK4*	*CDK6*	*CHN2*	*CREB3L2*	*CRLF2*
CTLA4	*CYB5R2*	*DENND3*	*DNMT3B*	*EGFL7*	*EPOR*	*ERG*
FLT1	*FLT3*	*FLT4*	*FOXP1*	*GPR110*	*HOXA9*	*ID4*
IGJ	*IL2RA*	*IRF4*	*ITPKB*	*JAK2*	*KDR*	*LIMD1*
LMO1	*LMO2*	*MAML3*	*MCL1*	*MECOM*	*MME*	*MUC1*
MUC4	*MYBL1*	*MYC*	*NRXN3*	*PAX5*	*PDGFRA*	*PIM2*
PTPN1	*RAB29*	*RARA*	*S1PR2*	*SEMA6A*	*SERPINA9*	*SH3BP5*
SOX11	*SPARC*	*SPATS2L*	*SPRED1*	*TAL1*	*TNFRSF13B*	*TP53INP1*
WT1						
